# Association between mitochondrial DNA haplogroups J and K, serum branched-chain amino acids and lowered capability for endurance exercise

**DOI:** 10.1186/s13102-022-00485-3

**Published:** 2022-05-26

**Authors:** Jukka M. Kiiskilä, Ilmo E. Hassinen, Johannes Kettunen, Laura Kytövuori, Ilona Mikkola, Pirjo Härkönen, Jari J. Jokelainen, Sirkka Keinänen-Kiukaanniemi, Markus Perola, Kari Majamaa

**Affiliations:** 1 Research Unit of Clinical Neuroscience, University of Oulu, P.O. Box 5000, 90014 Oulu, Finland; 2 Department of Neurology and Medical Research Center, Oulu University Hospital, Oulu, Finland; 3 Faculty of Biochemistry and Molecular Medicine, University of Oulu, Oulu, Finland; 4 Computational Medicine, Faculty of Medicine, University of Oulu, Oulu, Finland; 5 Center for Life Course Health Research, Faculty of Medicine, University of Oulu, Oulu, Finland; 6 Biocenter Oulu, University of Oulu, Oulu, Finland; 7 Finnish Institute for Health and Welfare, Helsinki, Finland; 8 Rovaniemi Health Center, Rovaniemi, Finland; 9 Unit of General Practice, Oulu University Hospital, Oulu, Finland; 10 Unit of Primary Health Care, Oulu University Hospital, Oulu, Finland; 11 Healthcare and Social Services of Selänne, Pyhäjärvi, Finland

**Keywords:** mtDNA haplogroup, Branched-chain amino acid, Trainability, Low-responder, Military conscript

## Abstract

**Background:**

Endurance exercise training promotes the catabolism of branched-chain amino acids (BCAAs) in skeletal muscles. We have previously shown that mitochondrial DNA (mtDNA) haplogroups J and K are markers of low responders in endurance training. In this paper, we hypothesize that BCAA catabolism is a surrogate marker of lower respiratory chain activity attributed to these haplogroups. We evaluated whether exercise-induced changes in amino acid concentrations differ between subjects harbouring mtDNA haplogroups J or K and those with non-JK haplogroups.

**Methods:**

Finnish male conscripts (N = 633) undertook the 12-min Cooper running test at the beginning and end of their military service. The intervention during the service mainly included endurance aerobic exercise and sports-related muscle training. Concentrations of seven amino acids were analysed in the serum using a high-throughput ^1^H NMR metabolomics platform. Total DNA was extracted from whole blood, and restriction fragment analysis was used to determine mtDNA haplogroups J and K.

**Results:**

The concentrations of the seven amino acids were higher following the intervention, with the exception of phenylalanine; interestingly, the increase in the concentrations of three BCAAs was larger in subjects with haplogroup J or K than in subjects with non-JK haplogroups (*p* = 0.029). MtDNA haplogroups J and K share two common nonsynonymous variants. Structural analysis based on crystallographic data on bovine complexes I and III revealed that the Leu18 variant in cytochrome b encoded by m.14798T > C may interfere with ubiquinone binding at the Qi site in complex III.

**Conclusions:**

The increase in the concentrations of serum BCAAs following exercise intervention differs between subjects harbouring mtDNA haplogroup J or K and those harbouring non-JK haplogroups. Lower response in endurance training and difference in exercise-induced increase in the concentrations of serum BCAAs suggest decreased respiratory chain activity. Haplogroups J and K share m.14798T > C in *MT-CYB*, which may hamper the function of complex III.

**Supplementary information:**

The online version contains supplementary material available at 10.1186/s13102-022-00485-3.

## Background

Skeletal muscle does not rely solely on carbohydrates and lipids in endurance exercise but also upregulates the oxidation of branched-chain amino acids (BCAAs) to support increased energy demands. The catabolism of BCAAs, i.e. isoleucine, leucine and valine, in skeletal muscle is promoted by endurance exercise training and down-regulated under sedentary conditions [1, 2]. Studies on experimental animals have shown that efficient use of BCAAs contributes to exercise capacity. Rats with enhanced endurance capacity show an improved energy metabolism where BCAAs and fatty acids are efficiently utilized as fuel during exercise [3]. Furthermore, a study on mice with a muscle-specific defect of BCAA catabolism has indicated that tight regulation of BCAA catabolism in muscle is important for adapting to exercise training [4].

Mitochondria are the site for BCAA metabolism [5], as most BCAA catabolic enzymes reside in mitochondria [6]. Accumulation of BCAAs has been observed in mitochondrial diseases [7] that are caused by pathogenic mutations in mitochondrial DNA (mtDNA) and that lead to impaired oxidative phosphorylation (OXPHOS) and reduced ATP production [8]. An increase in the concentrations of BCAAs has been observed in ρ^0^ cells, where OXPHOS has been eliminated by depleting the entire mtDNA [9]. Furthermore, inhibition of ATP synthase in mouse skeletal muscle cells leads to disturbances in the metabolic pathways of BCAA catabolism [10].

We have previously shown that the frequency of mtDNA haplogroups J and K is low among Finnish elite endurance athletes [11] and that Finnish military conscripts harbouring these haplogroups present with a lower response to exercise training [12]. Such findings suggest that mtDNA belonging to haplogroup J or K code for OXPHOS complexes that are less efficient in ATP production and hence contribute to poor endurance performance. These haplogroups are characterized by common variants in genes encoding subunits of complex I and III [13]. Interestingly, a significant increase in the concentrations of BCAAs has been found in *C. elegans* with a mutation in *gas-1*, an orthologue of human *NDUFS2* encoding a subunit of complex I or with a mutation in *isp-1*, an orthologue of human *UQCRFS1* encoding a subunit of complex III [14]. Here, we hypothesize that the BCAA metabolism is a surrogate biochemical marker of OXPHOS dysfunction and that subjects harbouring mtDNA haplogroup J or K differ from those harbouring non-JK haplogroups in their BCAA metabolism. To test the hypothesis, we measured the serum concentrations of BCAAs and four other amino acids in 633 young, healthy Finnish men undergoing a long-term exercise intervention during their compulsory military service. Furthermore, crystallographic data on bovine complex I and III were used to assess structural changes caused by the two common nonsynonymous variants that are shared by haplogroups J and K.

## Methods

### Subjects

In Finland, military service is compulsory for all men, and approximately 80% of them undergo training at the age of 19–20 years. The present study included 633 conscripts with a mean age of 19.17 years (SD 0.85; range 18–28 years) at the beginning of their service. The length of the service was 6 months for 456 conscripts (72.0%), 9 months for 28 conscripts (4.4%) and 12 months for 149 conscripts (23.5%).

Military service offers a unique opportunity to examine the effects of long-term exercise intervention on amino acid metabolism. During the training period, conscripts share similar living circumstances, and inter-individual variation is rather limited in terms of physical activity and caloric intake. This is due to the specific features of military service, such as strict discipline, mandatory physical training, institutional feeding and group living. All the conscripts follow the same daily schedule, including the time spent in exercise training. On average, physical training occupies approximately 450 h of the six-month service, i.e. 1040 min/week, consisting of sports-related physical activities—such as running, orienteering, cycling, cross-country skiing, Nordic walking and strength training—and military-related physical training, such as marching and combat training [15, 16]. The same prescribed diet is provided three times a day to all subjects during military service, but calorie intake is not strictly controlled. Approximately 75% of the energy intake during military service is covered by the meals served by the military forces [17].

### Molecular methods and clinical and physiological variables

At the beginning and end of military service measurements of body composition and anthropometry were performed [18] and aerobic performance was assessed using the standardized 12-min Cooper running test [19]. Serum samples were obtained after an overnight fast. The first sample was taken before the training began and the second sample was taken a few days after the training had ended. The serum concentrations of seven amino acids with metabolic pathways related to mitochondria [5] have been determined using a high-throughput ^1^H NMR metabolomics platform, as described elsewhere [20]. The amino acids included glutamine, glycine, phenylalanine, tyrosine, isoleucine, leucine and valine. The total concentration of BCAAs, i.e. the sum of isoleucine, leucine and valine concentrations, was calculated. The difference in the total BCAA concentrations measured at the beginning and end of the exercise intervention was referred to as dBCAA.

ABI Prism™ 6100 Nucleic Acid PrepStation with BloodPrep™ Chemistry Kit was used for the extraction of total DNA from whole blood (Applied Biosystems, Foster City, CA, USA). Mitochondrial DNA haplogroups J and K were identified using restriction fragment analysis [12].

### Molecular modelling

Haplogroups J and K share m.10,398 A > G in *MT-ND3*, and subhaplogroup J1 and haplogroup K share m.14798T > C in *MT-CYB*. Therefore, we evaluated the structural changes induced by p.Thr114Ala in subunit ND3 of complex I and p.Phe18Leu in subunit cytochrome b of complex III. Tertiary structure visualization and in silico mutagenesis of PDB files *5xtc* [21], *1ntz* [22] and *6zqm* [23] were performed using the Pymol 2.5 software running on Python version 2.7.12 (Schrödinger, LLC, available from www.anaconda.org).

### Statistical analysis

Statistical analysis was performed using the IBM® SPSS® Statistics Version 22 software. Non-parametric tests were used for statistical comparisons, as the distribution of most continuous variables was non-normal (Shapiro–Wilk, *p* > 0.05). Wilcoxon signed-rank test was used to evaluate how the exercise intervention changed the serum amino acid concentrations. Mann–Whitney U test was employed for comparing amino acid concentrations between subjects harbouring haplogroup J or K and those with non-JK haplogroups. The results are shown as medians and interquartile ranges.

Multiple linear regression analysis was carried out using dBCAA as dependent variable and the two groups of mtDNA haplogroups as independent variables. The changes in body mass index (kg/m^2^), body fat percentage, skeletal muscle mass (kg) and visceral fat area (cm^2^) were used as covariates. The dependent variable dBCAA was square-root transformed to produce a normal distribution. Spearman’s rank correlation was used to test for the association of total BCAA with the Cooper test distance at the beginning and end of the service. The correlation between dBCAA and the change in Cooper test result (dCooper) was also calculated. Furthermore, dBCAA was divided into quartiles, and the dCooper of the subjects in the top quartiles was compared with that of the subjects in the lowest quartile using Mann–Whitney U test. The lower rank was used for tied values. *P* values < 0.05 were considered to be statistically significant.

## Results

The conscripts (N = 633) took the 12-min Cooper running test, and their serum samples were analysed for the concentrations of seven amino acids at the beginning and end of their military training. The concentrations of the three BCAAs and three other amino acids increased, whereas that of phenylalanine remained constant (Table 1). There was an inverse correlation between the sum concentration of BCAAs and the Cooper test distance at the beginning (r = − 0.202, *p* = 2.99 × 10^− 7^; Spearman’s rank correlation test) and end of the service (r = − 0.126, *p* = 0.0015). The median increase in the Cooper test distance (dCooper) was 208.5 m during the military service, being 150 m in the highest quartile of dBCAA and 235 m in the lowest quartile of dBCAA (Mann–Whitney U test, *p* = 0.021). However, the correlation between dBCAA and dCooper was not significant (r = – 0.069, *p* = 0.083).

**Table 1:** Serum amino acid concentrations (µmol/l) in Finnish military conscripts (N = 633) at the beginning and end of military service

	Beginning	End	Change	*p* value*
Glutamine	290.0 (259.0–324.0)	350.0 (306.0–409.0)	60.0 (12.0–119.0)	1.6 × 10^− 42^
Glycine	272.0 (251.0–294.0)	276.0 (255.0–301.0)	3.0 (− 21.0 to 29.0)	1.6 × 10^− 2^
Phenylalanine	76.1 (70.2–82.9)	76.7 (71.9–82.9)	0.5 (− 6.9 to 7.3)	0.5
Tyrosine	51.6 (46.6–64.6)	57.1 (51.4–63.7)	4.9 (− 1.1 to 11.2)	3.0 × 10^− 31^
Isoleucine	57.2 (50.9–63.3)	68.5 (61.0–76.7)	12.0 (2.7–20.8)	5.8 × 10^− 68^
Leucine	84.8 (75.1–94.5)	94.2 (84.3–105.0)	9.9 (− 3.2 to 21.9)	1.4 × 10^− 30^
Valine	177.0 (159.0–197.0)	196.0 (174.0–216.0)	19.0 (− 6.0 to 43.0)	1.0 × 10^− 30^
Total BCAAs	320.0 (288.0–354.0)	360.0 (320.0–399.0)	41.2 (− 3.9 to 82.1)	4.8 × 10^− 42^

BCAA, branched-chain amino acid. The values are medians (interquartile range)

*Wilcoxon signed-rank test

In line with our previous study [12], which included 1036 conscripts, we found herein that the median improvement in the Cooper test was 102.4 m in conscripts harbouring mtDNA haplogroup J or K and 216.6 m in conscripts harbouring non-JK haplogroups (*p* = 0.0064, Mann–Whitney U test). Interestingly, the increase in the concentrations of all three BCAAs was higher among subjects harbouring mtDNA haplogroup J or K than among subjects with non-JK haplogroups (*p* < 0.05, Mann–Whitney U test, Table 2), whereas the increase in the concentrations of the remaining three amino acids was similar in the two groups. Furthermore, multiple linear regression analysis indicated a difference in dBCAA between subjects harbouring haplogroup J or K and those harbouring non-JK haplogroups after adjusting for covariates, such as the changes in body mass index, body fat, skeletal muscle mass, and visceral fat area (*p* = 0.027, Additional file 1: Table S1). The mean age of the subjects harbouring haplogroups J or K was 19.04 ± 0.71 years and that of subjects with non-JK haplogroups was 19.18 ± 0.86 years (*p* = 0.31 for difference). The frequency distribution of subjects who completed 6, 9 or 12 months of military service did not differ between subjects harbouring haplogroup J or K and those with non-JK haplogroups (2.773, *df* = 2, *p* = 0.250, X^2^-test). Further clinical characteristics of the groups are presented in Additional file 2: Table S2.

**Table 2:** Change in serum amino acid concentration (µmol/l) in conscripts with haplogroup J or K and in conscripts with non-JK haplogroups

	Haplogroups J and K	Non-JK Haplogroups	*p* value*
N	45	588	
Glutamine	92.0 (34.0–135.0)	63.0 (10.0–119.0)	0.058
Glycine	8.5 (− 18.5 to 32.5)	3.0 (− 21.0 to 27.0)	0.49
Phenylalanine	1.3 (− 5.2 to 11.2)	0.5 (− 6.9 to 7.2)	0.28
Tyrosine	4.3 (− 1.3 to 14.7)	4.9 (− 1.1 to 11.1)	0.60
Isoleucine	14.5 (6.2–30.4)	11.6 (2.4–20.1)	0.039
Leucine	15.6 (− 0.7 to 32.5)	9.4 (− 3.3 to 20.8)	0.045
Valine	30.0 (4.0–62.0)	18.0 (− 6.0 to 42.0)	0.035
Total BCAAs	69.7 (13.2–113.5)	39.9 (− 4.5 to 80.2)	0.029

BCAA, branched-chain amino acid. The values are medians (interquartile range)

*Mann–Whitney U test

**Fig. 1:**
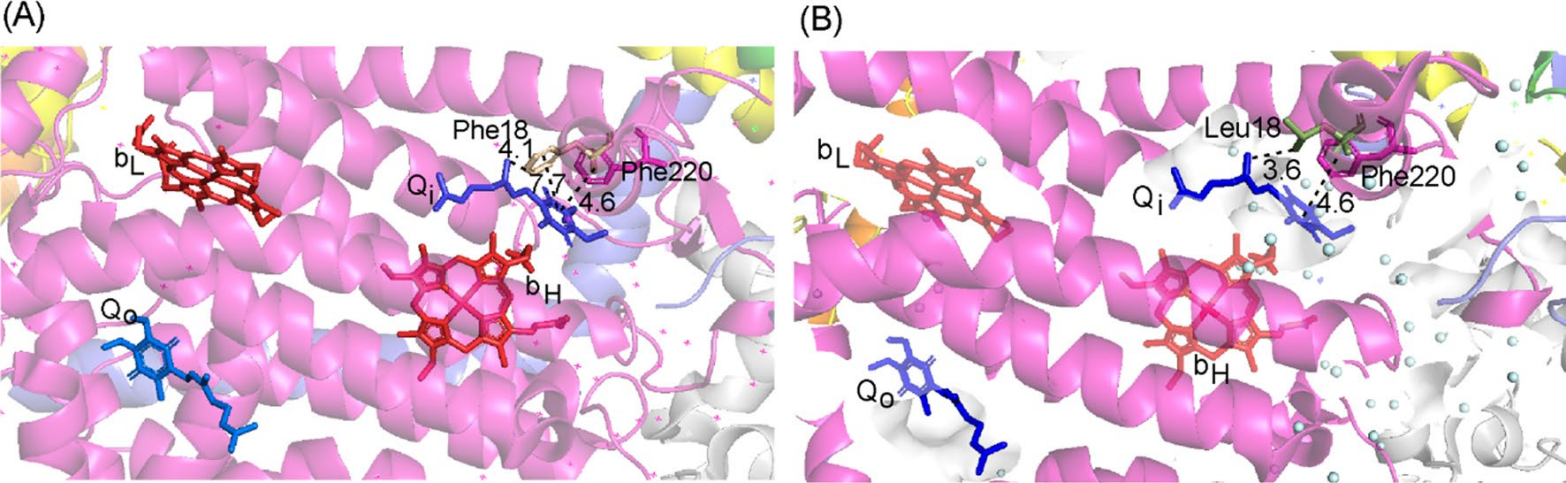
Mitochondrial ubiquinone-binding sites in **A** the reference and **B** the Phe18Leu variant cytochrome b. (Q_o_) intermembrane side ubiquinone; (Q_i_) matrix side ubiquinone; (b_H_) high potential heme b; (b_L_) low potential heme b. The distances are shown in ångströms. Both Phe18 and Phe220 are probably able to interact via π–π stacking with the quinol ring of ubiquinone Q_i_. An in silico Phe18Leu mutation in the coordinate file 1ntz.pdb and visualization were performed using the PyMol software

Tertiary structure visualization indicated that p.Thr114Ala is located in the penultimate position of the ND3 subunit in a flexible bend and appears not to interact with the nearest transmembrane helix of the ND1 subunit. The wild-type phenylalanine-18 in cytochrome b serves as a component of the wall of the ubiquinone-binding Q_i_ pocket. According to the bovine complex III structure coordinate file *1ntz.pdb* [22], Phe18 is close to the ubiquinone substrate bound in the Q_i_ pocket. The distance between the Phe18 ring and the 2,3-dimethoxy-5-methylbenzoquinone ring of ubiquinone is 7.7 Å and that between the Phe18 ring and the ubiquinone side chain is 4.8 Å. The side-chain methyl group of the Leu18 variant encoded in haplogroups J and K is only 3.6 Å from the ubiquinone side chain, enabling interaction with the substrate with a hydrogen bond (Fig. 1).

## Discussion

We found that the serum concentrations of all three BCAAs increased among healthy young men in response to a long-term exercise intervention. Interestingly, a large increase in total BCAA concentration was associated with low endurance performance and a low response to training. We have previously shown that mtDNA haplogroups J and K are infrequent among elite endurance athletes [11, 13] and that the two haplogroups are associated with low response to endurance training [12]. Here we found that the increase in BCAA concentration after the exercise intervention was higher among subjects with haplogroup J or K compared with subjects with non-JK haplogroups. This finding suggests that the mitochondrial respiratory chain affects the regulation of BCAA oxidation, but the mechanism remains elusive.

There is limited research addressing the question of whether long-term exercise affects resting plasma concentrations of BCAAs in humans. Our finding that the concentration of serum total BCAAs increased by 12.5% in young men during a rather standardized 6-, 9- or 12-month exercise intervention is partially in line with a study showing that the concentrations of leucine and valine are higher in trained university students than in untrained ones [24] as well as with another study showing that the concentration of resting plasma BCAAs increased in adults following progressive 20-week whole-body resistance exercise training [25]. An increase in BCAA metabolites has also been found in elite athletes in sports disciplines with high cardiovascular demand [26]. Furthermore, a study on athletic horses has shown that 12-week exercise training induces an increase in the concentration of resting plasma BCAAs [27]. It remains unclear how long-term endurance training leads to an increase in the plasma levels of BCAA, but studies on experimental animals subjected to short-term exercise training provide a possible explanation that would associate exercise-induced changes in BCAA concentrations with mitochondrial function [3, 14, 28].

Our finding on low endurance performance among subjects harbouring haplogroups J and K and experimental evidence from other studies indicate that these haplogroups code for mitochondrial respiratory chain complexes with a decreased capacity to produce ATP and hence contribute to redox imbalance. Indeed, experiments with rho^0^ cybrids harbouring mtDNA belonging to haplogroup J have revealed a lower NAD^+^/NADH ratio and lower ATP levels compared to rho^0^ cybrids harbouring haplogroup H mtDNA [29]. Furthermore, rho^0^ cybrids harbouring haplogroup J mtDNA or haplogroup K mtDNA are more sensitive to rotenone, a complex I inhibitor, compared with cells harbouring haplogroup H [30], suggesting a decreased OXPHOS capacity of these haplogroups. Healthy subjects with mtDNA haplogroup J also present with lower maximal oxygen consumption (VO_2max_) than subjects with non-J haplogroups [31]. None of the individuals with high VO_2max_ trainability in the HERITAGE family study harboured haplogroup J or K [32]. Moreover, these haplogroups have been found to be abundant among power and team sports athletes [11, 33, 34], whose performance relies more on anaerobic glycolysis rather than OXPHOS. Similar associations with mtDNA haplogroups have also been detected among Kenyan and Japanese athletes, as reviewed in [35].

We have previously shown that nonsynonymous mutational load consisting of common mtDNA variants is higher in subjects with haplogroup J or K than in subjects with other haplogroups [13]. Some of these variants could affect respiratory chain function, and here, we hypothesized that the changes in the BCAA catabolism in an exercise intervention are a surrogate of a respiratory chain defect attributed to haplogroups J and K. Interestingly, haplogroups J and K share two common nonsynonymous variants including m.10,398 A > G in *MT-ND3* encoding p.Thr114Ala; furthermore, subhaplogroup J1 and haplogroup K share m.14798T > C in *MT-CYB* encoding p.Phe18Leu in cytochrome *b* of complex III. Our analysis of crystallographic data from bovine complex I and III indicated that p.Thr114Ala appeared not to interact with the nearest transmembrane helix of the ND1 subunit, whereas p.Phe18Leu in cytochrome *b* appeared to affect the ubiquinone binding affinity of the *Q*_*i*_ site. Cytochrome *b* contains four redox active sites, including the high potential heme *b*_*H*_, the low potential heme *b*_*L*_ and the ubiquinone-binding site *Q*_*i*_ on the matrix side and *Q*_*o*_ on the intermembrane space side. These redox active components with the participation of the Rieske iron-sulphur protein and cytochrome *c*_*1*_ form the Q-cycle present a redox-driven transmembrane proton pump that functions in the conversion and conservation of the combustion energy of metabolic fuels. The p.Phe18Leu variant may thus affect the kinetics of the redox reaction and transmembrane proton pumping and compromise the mitochondrial aerobic capacity, thereby influencing endurance trainability. Indeed, mutations in *MT-CYB* are a possible cause of complex III deficiency and exercise intolerance in humans [36–38].

There is plenty of evidence showing that a decrease in mitochondrial respiratory chain activity is accompanied by an increase in BCAA concentration. Studies on *C. elegans* have shown that a mutation in *gas-1*, a gene encoding a 49 kDa subunit of mitochondrial complex I, leads to altered expression of biochemical pathways regulating BCAA catabolism and increases the whole-organism concentrations of BCAAs [14, 28]. Similarly, a complex I knockout induces a significant increase in the concentration of tissue BCAAs in the mouse [39]. *C. elegans* mutation in *mev-1* leading to complex II deficiency and the mutation in *isp-1* leading to complex III deficiency share a similar metabolic signature [28]. Furthermore, the burden of variants in *MT-CYB* has been associated with an increase in the concentration of circulating BCAAs in humans [40]. These findings, together with the fact that plasma BCAA is elevated in patients with a mitochondrial disease [7], suggest that mutations in genes encoding subunits of mitochondrial respiratory chain complexes may alter BCAA catabolism in humans. A decrease in respiratory chain activity leads to a decrease in the NAD^+^/NADH ratio, and it has been suggested that BCAA accumulation results from a lower activity of branched-chain α-keto acid dehydrogenase (BCKDH), which is dependent on NAD^+^ [39, 41]. Indeed, NAD^+^ is an important co-substrate in redox reactions and participates in the regulation of different metabolic pathways [42].

### Limitations of the study

We found that the response to training was lower and the increase in total BCAA concentration was larger following an exercise intervention in individuals harbouring haplogroups J or K compared with subjects with non-JK haplogroups. There are previous data showing that these haplogroups code for mitochondrial respiratory chain complexes with a decreased capacity to produce ATP [29, 30]. However, direct biochemical evidence is lacking that would connect exercise-induced changes to catabolism of BCAAs in these haplogroups and, unfortunately, we were not able to carry out such studies on mitochondrial biochemistry or exercise physiology on our study subjects. It should be also noted that the results of this study are only generalizable to healthy men of the age-group. It remains unclear, whether haplogroups J and K have similar association with serum BCAAs in women. Indeed, some of the variants in mtDNA that reduce male fitness, are neutral or beneficial in females and can reach high frequency in a population [43].

## Conclusions

This study shows that a long-term exercise intervention induces an increase in the concentration of serum BCAAs that is larger in subjects harbouring mtDNA haplogroup J or K than in subjects harbouring non-JK haplogroups. Lower response to endurance training and difference in exercise-induced increase in the concentration of serum BCAAs could be due to decreased mitochondrial respiratory chain activity. Indeed, haplogroups J and K share m.14798T > C in *MT-CYB*, which is absent in the remaining haplogroups. Molecular modelling showed that the m.14798T > C variant leads to structural changes in the cytochrome *b* subunit of complex III. A decrease in respiratory chain activity would slow down the mitochondrial catabolism of BCAA, and inefficient use of BCAAs as a fuel could partly explain the poor endurance performance attributed to mtDNA haplogroups J and K.

## Supplementary Information

**Additional file 1: Table S1**. Multiple linear regression model analysis with adjustment for various covariates. Multiple linear regression analysis indicates a difference in dBCAA between subjects harbouring haplogroup J or K and those harbouring non-JK haplogroups after adjusting for covariates (changes in body mass index, body fat, skeletal muscle mass, and visceral fat area).

**Additional file 2: Table S2** . Characteristics of the 633 conscripts in the beginning and in the end of the military service. Characteristics of subjects harbouring haplogroup J or K and those harbouring non-JK haplogroups.

## Data Availability

The data that support the findings of this study are available in this paper. Additional datasets generated during the study are available from the corresponding author on reasonable request.
